# Comparative Physicochemical and Electrochemical Characterization of the Structure and Composition of Thin Pd Binary and Ternary Codeposits with Pt, Ru, and Rh

**DOI:** 10.3390/ma11050798

**Published:** 2018-05-15

**Authors:** Katarzyna Hubkowska, Mariusz Łukaszewski, Michał Soszko, Urszula Koss, Bartosz Hamankiewicz, Andrzej Czerwiński

**Affiliations:** 1 Faculty of Chemistry, Warsaw University, Pasteura 1, 02-093 Warsaw, Poland; khubkowska@chem.uw.edu.pl (K.H.); ukoss@chem.uw.edu.pl (U.K.); bhamankiewicz@chem.uw.edu.pl (B.H.); 2 Industrial Chemistry Research Institute, Rydygiera 8, 01-793 Warsaw, Poland; michal.soszko@gmail.com; 3 Faculty of Chemistry, Biological and Chemical Research Centre, University of Warsaw, Żwirki i Wigury 101, 02-089 Warszawa, Poland

**Keywords:** hydrogen absorbing materials, metals and alloys, composition fluctuations, crystal structure, X-ray diffraction, photoelectron spectroscopies

## Abstract

Pd-Ru, Pd-Rh, Pd-Pt-Ru, and Pd-Rh-Ru electrodes were prepared as thin layers by potentiostatic codeposition or chemical co-precipitation of metals from baths containing mixtures of chloride salts. The formation of substitutional solid solutions, with lattice parameters smaller than that of pure Pd, was confirmed by X-ray diffraction (XRD). The compositions at various levels of sample volume and thickness were analyzed by inductively coupled plasma atomic emission spectroscopy (ICP-AES), energy-dispersive X-ray spectroscopy (EDS), X-ray photoelectron spectroscopy (XPS), Auger electron spectroscopy (AES), and also electrochemically by cyclic voltammetry (CV) in 0.5 M H_2_SO_4_. The differences between surface, subsurface, and bulk compositions were compared for various systems in a wide composition spectrum.

## 1. Introduction

Noble metal alloys, due to their good catalytic properties, have been widely examined as materials to be applied in electrochemical power sources. A known example is low-temperature fuel cells utilizing the oxidation reactions of hydrogen, methanol, ethanol, formic acid, and other organic fuels [1,2,3,4,5,6,7,8]. Palladium-based systems, as excellent hydrogen absorbers, are also particularly important in the context of hydrogen-related aspects of energy conversion and storage [9,10,11,12,13,14,15]. For the practical use, they can be considered as membranes for hydrogen purification/separation, components of hydrogen storage materials, electrode materials for electrochemical capacitors or hybrid cells, and also additives to Ni-MH cells that improve the operation of the hydride anode. On the other hand, in the fundamental research, they play a crucial role as the model representatives of the entire class of numerous hydrogen-metal systems [9].

It has been shown in the literature that an alloy electrode is usually a much better electrocatalyst than a given pure metal itself [16,17,18,19,20]. For instance, even platinum, which is a very active and efficient catalytic material, possessing unique electrochemical properties, cannot be used alone in low-temperature fuel cells [3,7,14,16,17,18,19,20,21,22,23,24]. Amongst the reasons, there are mechanistic issues concerning problems with Pt poisoning by the carbonaceous impurities present in hydrogen gas or by the intermediates formed during organic fuel oxidation [21,22]. In addition, the cathodic reaction of oxygen reduction is inhibited on pure Pt due to the formation of a thin layer of surface oxides that are difficult to reduce [23,24]. The application of binary, ternary, or quaternary alloys of Pt and other metals as the electrode materials often leads to a significant enhancement of the working parameters of the cells [16,17,18,19,20,25,26]. The beneficial effects result from an advantageous combination of different properties of various alloy components. The combination may lead to favorable changes in the electronic structure of the electrode itself (ligand model theory [21,27]), and/or to the alterations in the reaction mechanisms in the presence of different elements on the electrode surface (e.g., the bifunctional mechanism of methanol electrooxidation on Pt-Ru alloys [8,21,27,28,29]).

Due to the fact that the catalytic/electrocatalytic materials become more and more complex, it is crucial to have the ability to carry out their versatile characteristic in respect of various physical and chemical properties. This in particular concerns bulk properties, such as the electronic structure, crystal structure, and other material properties (e.g., elasticity), as well as the surface features [30,31,32,33,34,35]. The latter ones, although limited to the most external parts of the electrode, usually determine its electrocatalytic activity in a given reaction. All these features are dependent on the composition of an alloy, some of them on the entire bulk composition, and others on the surface composition, and are usually interrelated. Therefore, it is absolutely necessary to possess as much information as possible on the bulk and surface contents of the alloy components, which are often different, even in the case of homogeneous alloys [36,37,38,39]. The knowledge on the distribution of elements in the alloy bulk and on the surface, as well as the sample crystal structure, the degree of material homogeneity, or the presence of separate bulk/surface phases is also very important.

Different alloys composed of various elements, or even the same kinds of alloys of different compositions, often exhibit unique and selective catalytic behavior, in particular chemical/electrochemical reactions [40,41,42]. Moreover, materials which possess a high catalytic activity in a given process may be much more inert in another. Vice versa, the same reaction may proceed in a significantly different way in the presence of various catalysts. Thus, reproducible procedures of the preparation and further detailed characterization of various kinds of alloys are needed to fulfil the requirements of modern applied chemistry/electrochemistry/material science.

The alloy electrodes destined for electrocatalysis are synthetized in the form of either tight or incomplete coatings on conductive substrates, as well as monolayers, submonolayers, or islands of the size of micro-/nanometers. The methods of alloy electrode preparation include electrochemical codeposition, underpotential deposition, electroless deposition, chemical co-precipitation, vacuum deposition, volume melting, ball milling, powder sintering, and others [43,44,45,46,47]. The particular way of alloy preparation affects many properties of the obtained material, and the choice of a given synthesis method enables the control, to some extent, of the form, structure, morphology and composition of the electrocatalysts. Although each preparation method has its limitations, the application of various procedures of synthesis makes it possible to obtain catalysts possessing a variety of physical and chemical properties, suitable to various reactions.

Electrochemical codeposition of metals usually allows one to obtain alloy layers of a desired thickness, the properties of which depend on the composition of the electrolytic bath used, potential or current density applied, temperature, and also on the properties of the matrix chosen for the deposit [48,49]. While for technical and decorative purposes, very smooth, shiny, and wear resistant coatings are usually needed, most of the alloys designed for chemical industry and laboratory research should be characterized by a high surface roughness in order to ensure a great number of active surface centers for efficient catalysis.

Potentiostatic electrodeposition of multicomponent alloys requires applying a suitable potential, i.e., it should be low enough for reduction of each metal to occur, and the deposition rates of the metals should be similar. A steady-state electrocrystallization proceeds on the condition that the electron transfer process is much slower than the diffusion of metal cations to the electrode surface. Then the individual current densities of a particular cation reduction, and their concentrations at the electrode surface, are fixed during the deposition process, which leads to the formation of an alloy of a constant composition of the consecutively deposited layers [46].

The catalysts applied in chemical industry are usually in the form of fine powders, and are often synthetized by a chemical reduction of metal ions in a reaction with appropriate electron donors [49,50,51,52,53,54,55,56,57,58]. Noble metal nanoparticles deposited on carbon matrixes can be prepared by the following methods: (a) Direct reduction of metal complexes (e.g., with Cl^−^, CN^−^, NH_3_) in the presence of strong reducing agents (like NaBH_4_, hydrazine, or gaseous hydrogen), (b) colloidal method (like sol-gel method), (c) polyol method, (d) microemulsion method, and (e) core-shell nanoparticle synthesis.

In the present paper, we aim to summarize our results concerning the physical/chemical/electrochemical characterization of Pd-Rh, Pd-Ru, Pd-Pt-Ru, and Pd-Rh-Ru electrodes of different compositions, prepared in the form of limited volume electrodes by potentiostatic codeposition or chemical co-precipitation of metals from chloride baths. We have analyzed both recent and earlier experimental data obtained in our laboratory during several years of investigations on the electrochemistry of Pd binary and ternary alloys with Ru, Rh, and Pt. We make an attempt here to give a more detailed picture concerning surface, subsurface, and bulk structure and composition of various Pd-based systems.

## 2. Materials and Methods

Electrochemical experiments were performed at 298 K, in a three-electrode cell, using a Hg|Hg_2_SO_4_|0.5 M H_2_SO_4_ as the reference electrode and a Pt gauze as the auxiliary electrode. All potentials were recalculated with respect to the SHE.

The baths used for electrochemical alloy deposition were composed of mixtures of aqueous solutions of PdCl_2_, H_2_PtCl_6_, RhCl_3_, RuCl_3_, and HCl. Different compositions of the deposits could be obtained by changing deposition potential and bath composition. Pd-Pt-Ru samples were also obtained as nanoparticles on carbon support by a chemical reduction (using NaBH_4_) of chloride salts dissolved in water suspension containing Vulcan XC72R carbon nanopowder (supplied by Cabot, Boston, MA, USA) [59,60]. All alloy compositions are expressed in atomic percentages.

The surface morphology of the samples was examined using scanning electron microcopy (SEM). Alloy bulk compositions were determined by inductively coupled plasma atomic emission spectroscopy (ICP-AES) and energy-dispersive X-ray spectroscopy (EDS). Structural characterization of the electrodes was performed by X-ray diffraction (XRD). Alloy surface/subsurface state and compositions were characterized by X-ray photoelectron spectroscopy (XPS), Auger electron spectroscopy (AES), and also electrochemically by cyclic voltammetry (CV) in 0.5 M H_2_SO_4_ aqueous solutions.

For SEM and EDX measurements, a Merlin scanning electron microscope (Zeiss, Oberkochen, Germany) was used together with a Quantax 400 energy dispersive X-ray spectroscope (Bruker, Billerica, MA, USA), or a Nova NanoSEM 450 (FEI, Hillsboro, OR, USA) coupled with an EDAX Apex/Genesis XM 2 energy dispersive X-ray spectroscope. Electron beam energy used for X-ray excitation was 10–15 keV and the spectrum acquisition time was ca. 100–120 s. For quantitative EDX data processing, a standardless procedure was applied with the use of the software supplied by Bruker. For energy calibration, X-ray signals obtained from pure Cu were utilized.

XRD patterns were collected using classical Bragg–Brentano focusing geometry by D5000 (Bruker AXS) diffractometer equipped with a Cu X-ray tube (40 mA, 40 kV) and a Ni filter (1:20). A Lynx Eye (Bruker AXS) detector with 192 measuring LEDs was used.

XPS spectra were collected by Microlab 350 (Thermo Electron, VG Scientific, Waltham, MA, USA). High resolution spectra were measured with pass energy of 23.5 eV from 250 µm × 250 µm areas of the samples with a resolution of 0.83 eV using the binding energy of carbon (C1s: 285.0 eV) as the reference. X-ray monochromatic radiation of 1486.6 eV (Al_Kα_, 25 W) was used. The pressure during analysis was 5.0 × 10^−9^ mbar. A linear or Shirley background subtraction was applied to obtain XPS signal intensity. The peaks were fitted using an asymmetric Gaussian/Lorentzian mixed function.

ICP-AES analyses were performed at Analytical Laboratory of Polish Mint.

## 3. Results and Discussion

### 3.1. Structural Characterization

In order to examine the crystal structure of Pd-based deposits, and to verify the possibility of an alloy formation during metal codeposition, XRD measurements have been performed for the freshly prepared samples. The spectra were obtained under various conditions, namely: (1) First in air atmosphere, (2) in vacuum, (3) in He, (4) in H_2_ gas, and (5) back in H [58].

**Figure 1:**
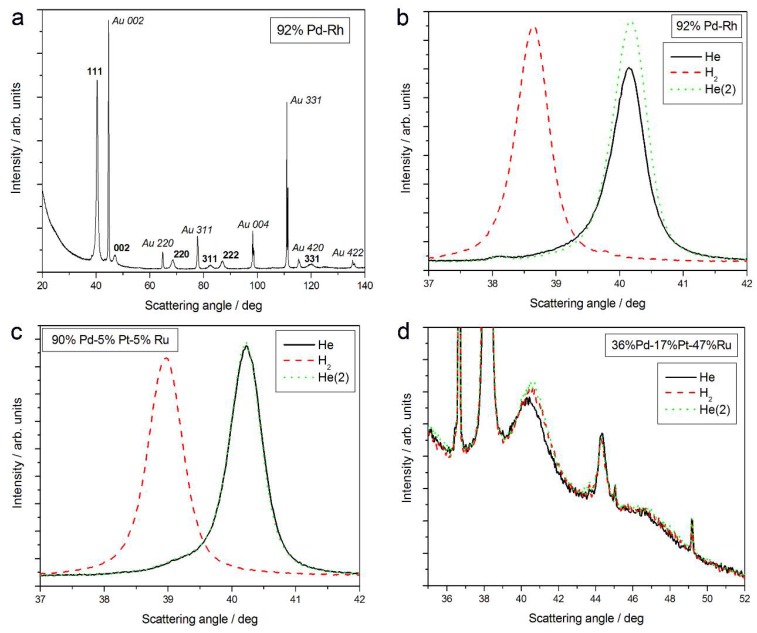
X-ray diffraction (XRD) patterns of Pd-based alloys of different bulk compositions: (**a**) 92% Pd-Rh, full spectrum in air; the evolution of spectra in the region of (111) signal in He; H_2_ and again in He for: (**b**) 92% Pd-Rh; (**c**) 90% Pd-5% Pt-5% Ru; and (**d**) 36% Pd-17% Pt-47% Ru.

Figure 1a shows an XRD pattern obtained in air in a wide range of scattering angles (2 theta = 20–140°) for a freshly deposited Pd-Rh electrode (92 at % Pd in the bulk), not subjected to any kind of pretreatment. Two series of lines can be distinguished in the spectrum, namely those originating from the deposit and from the Au substrate. For both parts of the sample, the reflection angles from various crystal planes (111, 002, 220, 311, 222, 004, 331, 420, and 422) of face-centered cubic (fcc) crystal lattices were identified, as indicated in Figure 1a.

The evolution of 111 signal in different atmospheres is presented in Figure 1b. When the Pd-Rh sample is exposed to gaseous hydrogen, the signal is shifted negatively, and it returns to its initial position again under helium atmosphere.

The analysis of XRD data for 111 signal reveals that in the case of fresh deposits, the lines other than those attributed to Au are placed at scattering angles intermediate between the values typical of pure Pd (2 theta = 40.153°) and Rh (2 theta = 41.075°). The values of lattice parameters for Pd and Rh are 3.891 Å and 3.803 Å, respectively, while for the Pd-Rh sample, the analyzed lattice parameter was determined as 3.884 Å [31,32,58]. This is evidence of the formation of a homogeneous substitutional solid solution, i.e., a Pd-Rh alloy, during Pd and Rh codeposition from the bath used. A smaller lattice parameter of the alloy than that of Pd indicates the crystal lattice contraction upon alloying (contracted alloy) [31]. Any additional signals that could be attributed to pure Pd, Rh, or other metals/phases (except for underlying Au) are not visible. This means that the Pd-Rh deposit consists of a single alloy phase.

A similar behavior was observed for other Pd-rich binary and ternary electrodeposits containing Ru and/or Rh, where contracted alloys were also obtained [58]. This situation is in line with the fact that the Ru-Ru distance in the hexagonal close-packed (hcp) structure gives the calculated lattice parameter for fcc symmetry = 3.827 Å and the scattering angle (2 theta) = 40.858°, i.e., indicating a smaller size of the unit cell. In contrast, for pure Pt, the values of lattice parameter and 2 theta are 3.924 Å and 39.798°, respectively, indicating a greater size of the unit cell compared with Pd [31,32,58]. However, for the range of bulk compositions of the Pd-Pt-Ru alloys studied here, the effect of the crystal lattice contraction due to the presence of Ru dominated over the opposite effect of the crystal lattice expansion due to the presence of Pt. In that context, it should be mentioned that although Pd-Pt binary alloys are known as expanded systems, their behavior in the process of hydrogen absorption exceptionally resembles that typical of contracted alloys [61,62,63].

**Figure 2:**
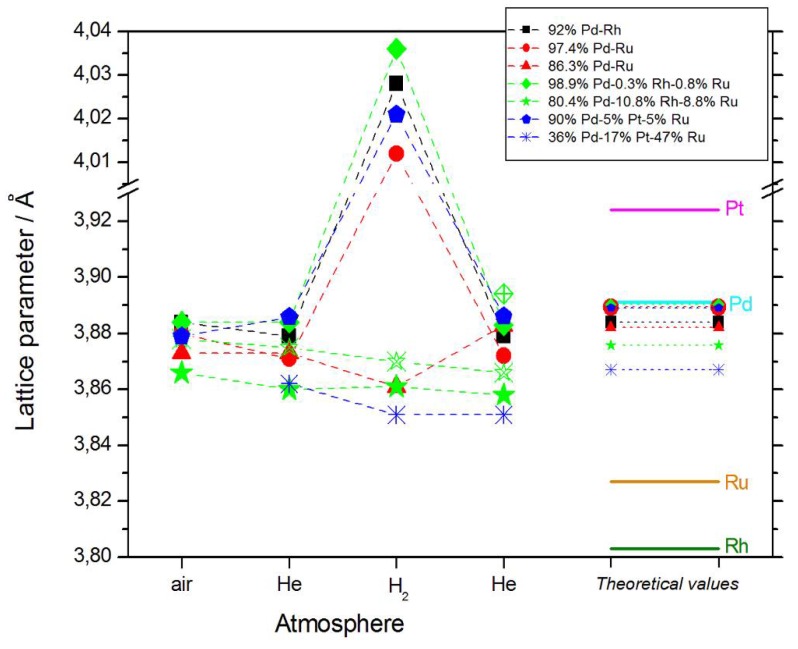
The values of lattice parameter calculated from XRD spectra obtained for samples of different elemental compositions in various atmospheres, together with the values for pure metals (solid lines), and predicted for the alloys on the basis of Vegard’s law (dotted lines). Note that for both Pd-Rh-Ru samples, two phases exist of different lattice parameters, which is indicated by the solid and open symbols.

The values of lattice parameter for samples of different elemental compositions, calculated from XRD spectra obtained at various stages of the described experiments, are collected in Figure 2. These data can be summarized in the following ways:

(1) In most cases, lattice parameters of fresh deposits are close to those predicted by Vegard’s law;
(2) Under hydrogen atmosphere, the lattice parameter for Pd-rich samples considerably increases, while for the samples poorer in Pd, it almost does not alter, or even slightly decreases;
(3) Lattice parameter in vacuum or in helium before or after exposure to hydrogen is often slightly smaller than that in air.

These points require more detailed comments [64]:

(1) The good agreement with Vegard’s law suggests that the alloy phase is the only one present in the samples, i.e., that the deposits are highly homogeneous. However, the literature data show that such a conclusion should be drawn with care (see Januszewska et al. [65] and the references therein). In particular, there are evidences that even in homogenous systems, the deviations from Vegard’s law are observed, and vice versa, Vegard’s law can be very well fulfilled in the case of heterogeneous materials.
(2) The magnitude of changes in lattice parameter under hydrogen atmosphere depends on the fact whether a given sample can absorb hydrogen or not. This is illustrated in Figure 1c,d, comparing XRD patterns for two Pd-Pt-Ru samples with different abilities to absorb hydrogen. While for a Pd-rich (ca. 95% Pd in the bulk) Pd-Pt-Ru sample, lattice parameter increases under hydrogen atmosphere and the diffraction lines are shifted negatively, in the case of the sample containing ca. 36% Pd in the bulk, no expansion of the crystal lattice occurs, as mirrored by no alterations of XRD spectrum. The constant position of signals in XRD spectra during the exposure to gaseous hydrogen is typical of those Pd-based deposits which contain too little Pd in the bulk for hydrogen absorption in the β-phase to occur [9]. In the presence of only negligible amounts of hydrogen absorbed in the α-phase, the lattice parameter increases very weakly in comparison to that of a non-hydrogenated material [31,32], and therefore its slight change is not detected by XRD. It should be added that lattice parameter for the hydride phase in the Pd-Rh alloys containing ca. 92 at % Pd in the bulk (4.028–4.043 Å) is even greater than that for Pd hydride (4.025 Å), which is consistent with the literature [31].
(3) The small decrease in lattice parameter after pumping out the air and then after contact with hydrogen could indicate changes in the composition of the alloy phases. For instance, using Vegard’s law, one obtains the alloy bulk composition as ca. 92% Pd-Rh for the fresh alloy, and ca. 86% Pd-Rh after hydrogen absorption/desorption. The former value is in a very good agreement with atomic emission spectroscopy data. The small decrease in lattice parameter, converted via Vegard’s law into the apparent alloy enrichment with Rh, can be explained taking into account the following possibilities [64]: (i) An irreversible surface segregation accelerated by superabundant vacancy formation (SAV) [66] that may accompany hydrogen absorption, and (ii) an additional reduction of small amounts of Rh and/or Ru surface oxides (present on the surface after contact with the deposition bath) to the metallic form by atomic hydrogen taking part in the absorption process.

For the deposits containing greater bulk amounts of Ru, two additional types of behavior have been observed. Ru is characterized by a different crystal structure (hcp) than Pd, Rh, and Pt (fcc). First, bulk heterogeneity may appear due to the phase segregation originating from the presence of a miscibility gap in the phase diagrams of the binary or ternary systems at high Ru contents [67,68]. Such a situation was observed in case of Pd-Rh-Ru and Pd-Pt-Ru deposits [58]. Moreover, hydrogen absorption can also force the process of phase separation in Pd-rich samples [64,66]. Second, a type other than fcc symmetry was determined for Ru-rich (>46% in the bulk) Pd-Pt-Ru electrodes, where icosahedral geometry was suggested. These effects are discussed in a separate report [58].

Moreover, even for the homogeneous Ru-rich (>37% in the bulk) samples, a partial segregation within individual crystallites is highly possible. This conclusion is based on the observed systematic deviations from Vegard’s law, suggesting a non-homogeneous distribution of the elements in the crystallites. In fact, the position of XRD signal originating from a given phase characterized by a given lattice constant is more strongly affected by the composition of the crystallite interior than that of the outer layers [64]. Therefore, a metal concentration gradient inside the crystallites is manifested in the determined value of lattice parameter different from that expected, in the case of true homogeneous crystal domains. The XRD data obtained for Ru-rich Pd-Pt-Ru deposits indicate an excess of Ru or Pt in the crystallite core at the expense of other metals, which dominate near the crystallite surface [58,64].

Similar structural features are exhibited by Pd-Pt-Ru materials obtained by chemical reduction. The main difference between the electrochemically codeposited and chemically coprecipitated Pd-Pt-Ru samples concerns the crystallite size. In the latter case, there is a relatively wide distribution of the parameter values, with the presence of ca. 10% fraction of very small crystallites (below 2 nm) and the weighted average crystallite size ca. 9–16 nm. On the other hand, the electrodeposits are composed of the crystallites of a much more uniform size, although with a smaller mean value (4–6 nm). The fraction of the finest crystallites tends to undergo oxidation when exposed to air.

It should be added that some differences in morphology were observed between the electrochemically deposited and chemically co-precipitated Pd-Pt-Ru samples. SEM inspections of the electrode surfaces revealed that all fresh Pd-Pt-Ru electrodeposits fully covered the underlying Au substrate, i.e., neither cracks nor naked areas could be observed [58]. Such tight coatings were also obtained for other types of electrodeposited alloys, i.e., Pd-Pt, Pd-Ru, Pd-Rh, Pd-Pt-Rh, and Pd-Rh-Ru. On the other hand, in the transmission electron microscopic (TEM) images obtained for Pd-Pt-Ru/VulcanXC72 samples prepared by chemical reduction, the areas of low and high concentrations of the metallic particles could be distinguished. The size of the particles ranged from ca. 2.5 nm to 7 nm, and some parts of the samples were covered by agglomerated particles, forming a layer of a thickness of tens of nanometers, but not greater than ca. 50 nm. Bare support areas, not covered by the metals, were visible as well.

The small size of metal particles is favorable in the context of catalytic applications due to a high surface development, providing a large number of active centers per mass unit. However, the presence of naked substrate regions is disadvantageous, as a possible source of problems with conductivity and transport of the reacting substances during electrode operation in fuel cells. The fact that both nanoparticles and agglomerates are visible on the samples indicates a considerable degree of morphological inhomogeneity of the catalysts in the micro-scale. This may cause difficulties in the reproducible preparation of the electrodes of similar properties, and in the determination of their real surface areas.

### 3.2. Characterization by Spectroscopic Techniques

Pd-based electrodeposits were characterized by such spectroscopic techniques as energy dispersive X-ray spectroscopy (EDS), X-ray photoelectron spectroscopy (XPS), and Auger electron spectroscopy (AES; note not to confuse this acronym with atomic emission spectroscopy, which is denoted here as ICP-AES). In the following presentation and discussion of data, Pd-Rh electrodes will often be described as representative systems for the physicochemical characteristics of Pd-based deposits, as in many cases other samples studied exhibited similar properties, and therefore the data obtained for the Pd-Rh system could serve as a basis for a general discussion. However, since various Pd-based alloys also possess some unique properties limited to certain surface and bulk compositions, such cases will be pointed at, and the differences will be emphasized, when discussing data for the particular samples.

**Figure 3:**
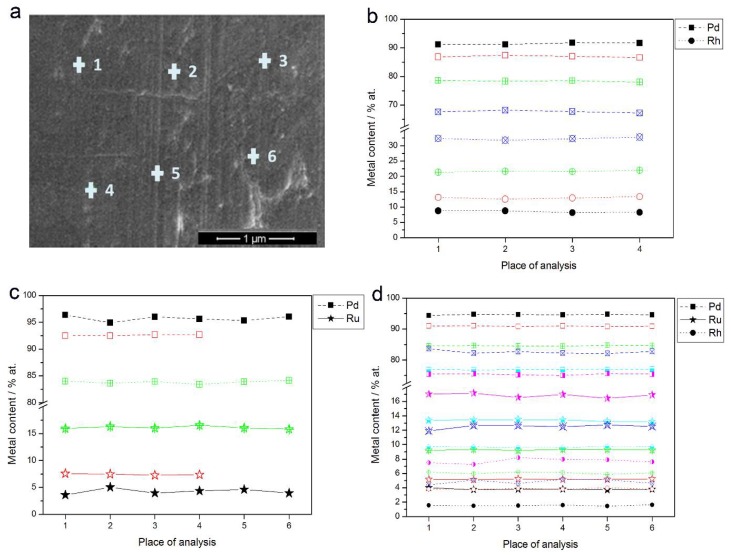
(**a**) SEM image of a Pd-Rh alloy (ca. 92% Pd in the bulk); (**b**–**d**) the results of local energy-dispersive X-ray spectroscopy (EDS) analyses of Pd-based alloys of different bulk compositions. Each colored line corresponds to a given alloy with metal contents represented by the symbols indicated in the legend.

Figure 3a shows an SEM image of a Pd-Rh electrode (ca. 92% Pd in the bulk) with the indicated points of local X-ray microanalyses performed by the EDS technique. The obtained local compositions of various Pd-Rh, Pd-Ru and Pd-Rh-Ru samples are collected in Figure 3b–d. Due to the fact that for this method the analytical signal originates from a thickness of ca. 0.5–1.0 μm [69,70], the obtained data concern bulk compositions of the deposits, whose thickness is also of this order of magnitude.

The differences in metal concentrations from place to place chosen for EDS analysis do not exceed ±1 at %. The results indicate that within the volumetric scale corresponding to X-ray escape depth, and with a lateral resolution of a similar dimension, the compositions of the electrodeposited samples are uniform.

However, EDS analyses performed at various parts of even many samples do not give the complete information on the degree of homogeneity of the electrodes obtained, since it is possible that despite very similar total bulk compositions, the metal contents in the particular layers may be different. This is especially likely for the surface and subsurface parts of the sample, due to the presence of a surface excess of a given element with respect to the alloy bulk. However, these possible differences become averaged in the EDS signal.

**Figure 4:**
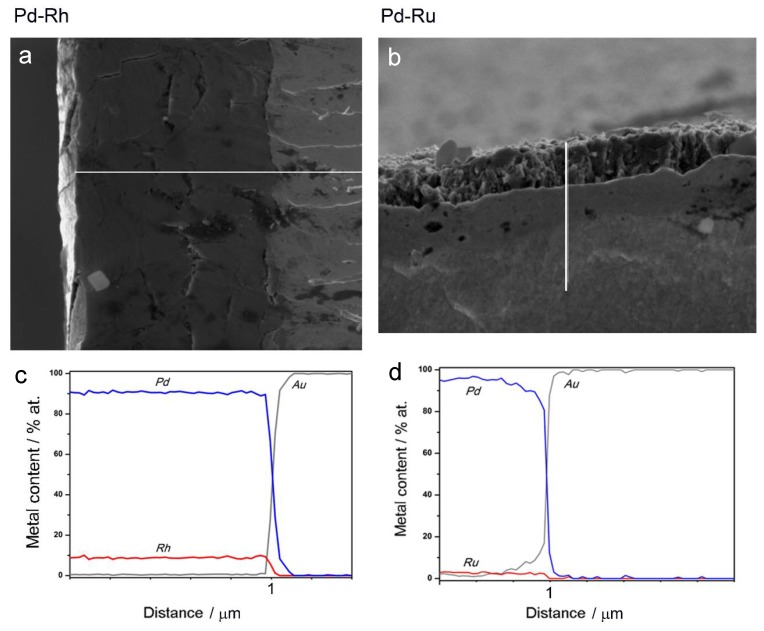
SEM images and EDS analyses of cross-sections of fractured Pd-Rh (**a**,**c**) and Pd-Ru (**b**,**d**) samples. White lines correspond to the length of the depth profiles.

Therefore, another kind of experiment was performed, concerning EDS analysis along a line perpendicular to the surface for the samples, which had been fractured after 3 h cooling in liquid nitrogen. Thus, it was possible to obtain depth profiles of the elements in the samples studied. Figure 4a,b show SEM images of the fractured Pd-Rh and Pd-Ru samples, respectively, and Figure 4c,d present the results of EDS analyses along the white lines indicated on the images.

Figure 4 clearly demonstrates the presence of a sharp boundary between the deposited layers and Au matrixes, visible not only on SEM images, but also mirrored in a rapid drop in Pd and Rh or Ru concentrations, accompanied by an abrupt increase in Au concentration from 0 to 100%, in very narrow sections of the profiles. On the other hand, following a line from the surface to the deposit/substrate boundary, the concentrations of Pd and Rh or Ru within the deposit volume alter weakly, which suggests a high degree of homogeneity of successive atomic layers of the alloy. Again, one should note that these results concern the depth and lateral dimensions of the order typical of EDS technique, i.e., ca. 0.5–1.0 μm [69,70]. Thus, the sample compositions obtained for various points along a chosen line are averaged over the neighboring areas/volumes around each point, and also over many deposited layers (also corresponding to a thickness of 0.5–1.0 μm) lying in the bulk below each of these surface points. Therefore, the conclusion on the composition homogeneity of the Pd-Rh and Pd-Ru deposits, drawn on the basis of the data presented in Figure 4, is still justified within the framework of the limitations of the EDS technique.

Another technique applied for the analysis of the composition of the deposits was XPS. Here, the information depth is ca. 10 nm, i.e., corresponding to tens of atomic layers beneath the surface [69,70]. Thus, the results of XPS analysis are much more affected by surface effects, and are independent of the composition of most of the sample volume. Therefore, the possible deviations in surface compositions with respect to the bulk can be manifested in the XPS signal. In this technique, the deconvolution of signals additionally enables the determination of the chemical state of the elements [71].

In the case of freshly prepared Pd-Ru deposits, XPS analysis has indicated the presence of both metallic and oxidized forms of the elements. Pd oxides have been identified as PdO, while Ru oxides as a mixture of RuO_2_, RuO_3_, and RuO_4_. Traces of Pd and Ru compounds with Cl atoms were also detected, probably due to residual PdCl_2_ and RuCl_3_ salts remaining on the surface from the deposition bath. Similar chemical states of Pd and/or Ru were also observed in the case of Pd-Rh and Pd-Rh-Ru alloys, where Rh was identified both in its metallic form and as Rh_2_O_3_.

**Figure 5:**
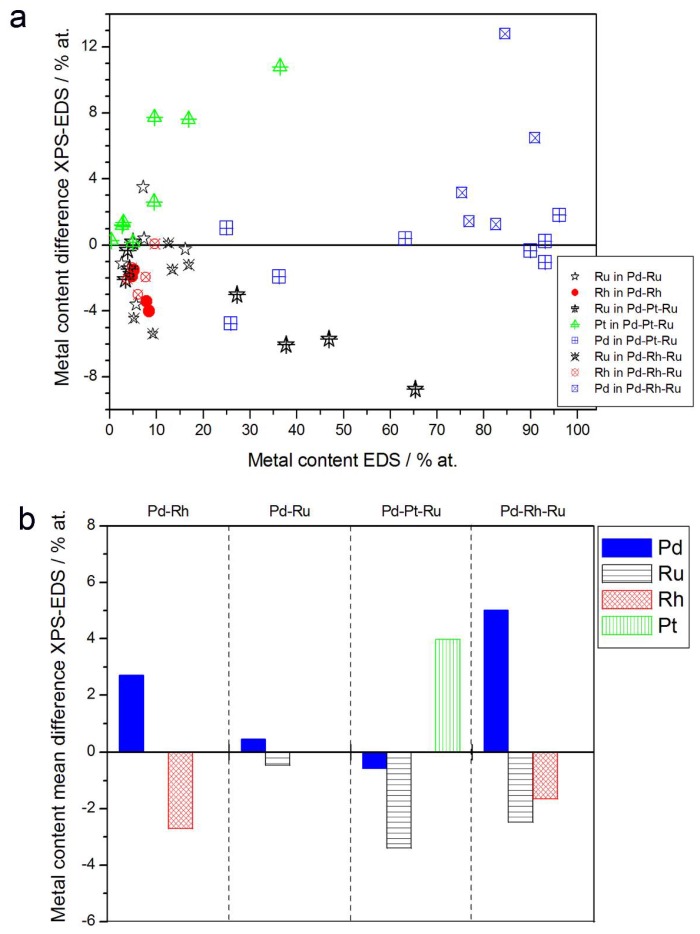
Comparison of the results of quantitative X-ray photoelectron spectroscopy (XPS) and EDS analyses of Pd alloys with Ru, Rh, and/or Pt. (**a**) Difference between metal concentrations determined by XPS and EDS versus EDS-derived concentrations for individual samples. (**b**) Mean difference between metal concentrations determined by XPS and EDS for each type of alloy.

Figure 5 presents the comparison of the metal concentrations in various samples determined by the XPS and EDS techniques. The analysis of those data reveals the following facts:

(1) In the case of Pd-rich (>85% Pd in the bulk) binary Pd-Ru deposits, it is difficult to find a distinct, unequivocal tendency in the relation between bulk and surface/subsurface content of the metals. For individual Pd-Ru samples, a rather large scatter of data is observed within ±4%, and the mean difference between the metal contents derived from XPS and EDS does not exceed ±0.5 at %;
(2) In case of Pd-rich (>90% Pd in the bulk) binary Pd-Rh deposits, XPS detected ca. 1.5–4% less Rh than EDS (a difference mean value ca. −2.7 at %);
(3) A systematic excess of Pd content in XPS vs. EDS data (a difference mean value ca. +5 at %) at the expense of Rh and Ru is observed in the case of Pd-rich (>80% in the bulk) Pd-Rh-Ru deposits;
(4) Ru content determined from XPS data is smaller than that obtained by EDS for Pd-Pt-Ru and Pd-Rh-Ru electrodes. For individual Pd-Pt-Ru samples, the depletion with Ru visible by XPS becomes greater with increasing Ru bulk content, reaching ca. −8.7 at % for a sample containing 65% Ru in the bulk. The mean differences between both sets of data are ca. −3.7 at % (and almost −6 at % for Pd-Pt-Ru samples containing more than 25% Ru and less than 65% Pd in the bulk) and −2.5 at %, respectively;
(5) In the case of Pd-Pt-Ru samples, much more Pt is indicated by XPS than by EDS, i.e., by up to +11 at % individually and ca. +4 at % on the average (and even +6 at % for Pd-Pt-Ru samples containing less than 65 at % Pd in the bulk).

In order to discuss whether these differences have a relevant physical meaning, it should be taken into account that, in general, for bimetallic or multicomponent solid systems, the surface/subsurface composition can differ from the bulk composition due to many possible reasons, such as thermodynamic tendency to a surface excess of one element, or a particular method of sample preparation and the procedure of further treatment.

First, we will consider the case of Pd-Rh electrodes. According to the literature, data for the Pd-Rh system [72,73], homogeneous Pd-Rh alloys under conditions of thermodynamic equilibrium are superficially enriched with Pd, while for Pd-Rh electrodeposits, the surface is enriched with Rh (for the samples containing more than ca. 20% Rh in the bulk), or no significant differences in its composition with respect to the bulk are observed (for the samples very rich in Pd).

**Figure 6:**
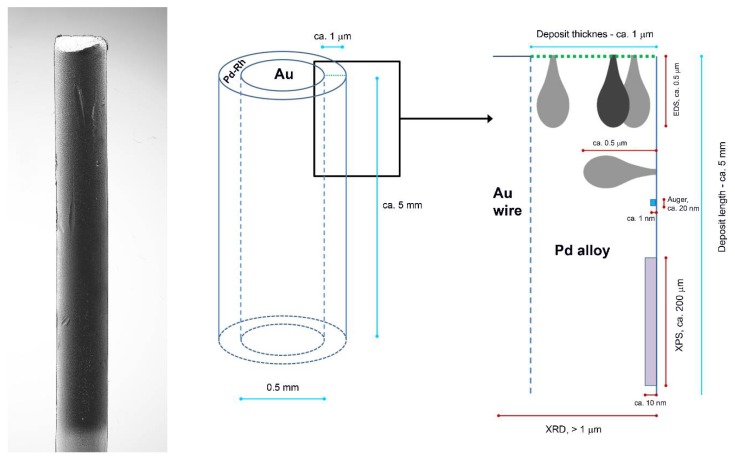
Scheme of Pd-based thin layer deposited on Au wire and the order of information depth provided by various analytical techniques applied in this study (based on the idea by Janik-Czachor and Pisarek [70]).

Although our results of XPS analysis for the same Pd-Rh deposits gave Pd concentration by ca. 1.5–4% greater than that given by EDS, this small surface/subsurface excess of Pd suggested by XPS data is not manifested in EDS profiling experiment, where Pd concentration at the very first point near the surface does not deviate from the values for deeper atomic layers. This can be explained by the aforementioned differences in the information depths and lateral resolutions of EDS/XPS analyses. These features of various analytical methods are schematically depicted in Figure 6, which is based on the idea presented by Janik-Czachor and Pisarek [70].

In particular, the dimensions of the surface and subsurface regions corresponding to the initial points of the line chosen for profiling are relatively small, in comparison with the surface and subsurface area/volume characterized by an XPS measurement. In the latter case, the X-ray beam penetrates into the sample and the photoelectrons escape from a large portion of the sample, if one considers the area exposed to the exciting beam (ca. 250 μm × 250 μm). On the one hand, regarding the size of the incident beam zone, the information on the composition of the most outer surface layers obtained by EDS profiling is more local compared to XPS data. However, on the other hand, EDS results are also affected by the possible composition fluctuations in a volume of the order of ca. 1 μm^3^ where X-rays are excited, involving underneath and neighboring subsurface layers, which is in contrast to a shallow escape depth of the photoelectrons [69,70].

The above results confirm the earlier statement on the sample composition homogeneity in scales characteristic of the resolution of these two analytical methods. However, in order to characterize the true surface state of the samples, another spectroscopic technique should be used, which is more sensitive to surface composition and enables much more local analysis. Such a condition is fulfilled by AES, where the lateral resolution reaches ca. 20 nm or even less, and the information depth is of the order of 1 nm, i.e., it corresponds to only a few external atomic layers [69,70].

**Figure 7:**
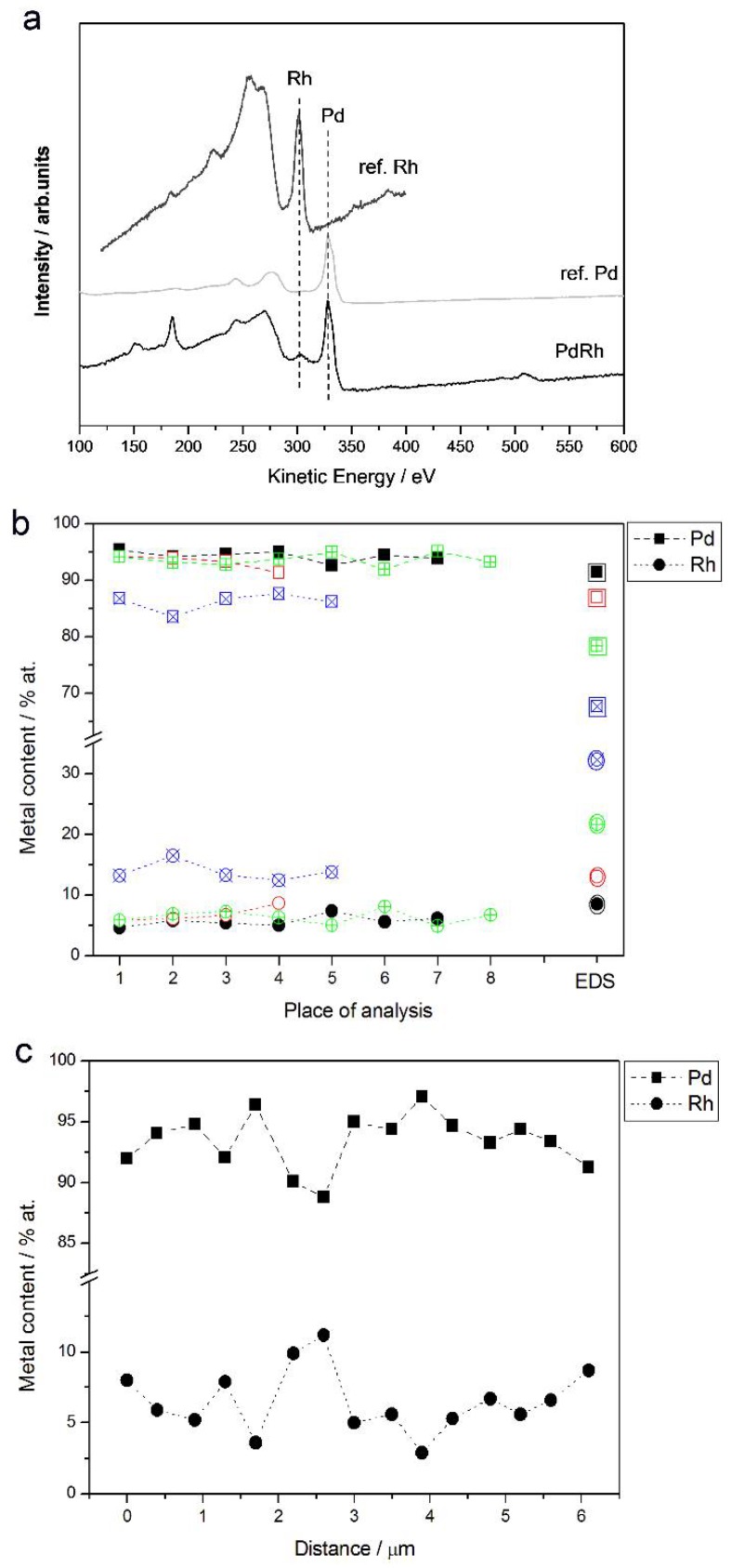
(**a**) Auger electron spectrum for a Pd-Rh alloy (92% Pd in the bulk); (**b**) the results of local Auger electron spectroscopy (AES) analyses of various Pd-Rh samples compared with EDS data; (**c**) linear profiling AES data of a Pd-Rh sample (87% Pd in the bulk). Each colored line corresponds to a given alloy with metal contents represented by the symbols indicated in the legend.

Figure 7a shows an example of an Auger electron spectrum for a Pd-Rh deposit (ca. 92% Pd in the bulk), together with the reference spectra for pure metals. The results of AES analyses from selected regions with a lateral resolution of ca. 20 nm are collected in Figure 7b. For a comparison, the compositions of the same samples analyzed by EDS are shown. AES analysis was also performed along a line segment across a surface with a total length of ca. 6 μm and with a lateral sampling interval of ca. 400 nm. The results of that experiment are presented in Figure 7c.

**Figure 8:**
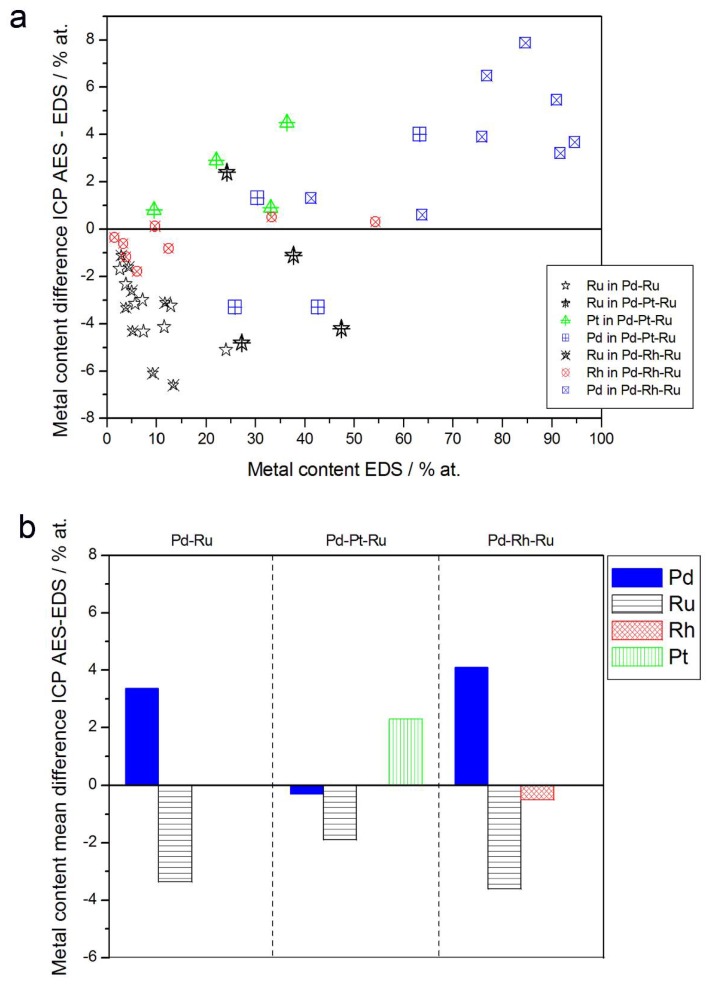
Comparison of the results of quantitative inductively coupled plasma atomic emission spectroscopy (ICP-AES) and EDS analyses of Pd alloys with Ru, Rh, and/or Pt. (**a**) Difference between metal concentrations determined by XPS and EDS versus EDS-derived concentrations for individual samples. (**b**) Mean difference between metal concentrations determined by XPS and EDS for each type of alloy.

As visible in Figure 8b, some fluctuations in Pd and Rh concentrations are observed between consecutive analysis points, these deviations are greater (up to ca. ±2.5 at %) than in case of EDS measurements, and a relatively high degree in the composition homogeneity is still observed. One should remember that the conclusion on the deposit homogeneity concerns a few of the most outer atomic layers of the samples and a lateral resolution of a local AES analysis, i.e., of the order of several tens of nanometers.

For Pd-Rh deposits very rich in Pd in the bulk (>90%), the compositions determined by both EDS and AES are very close (within ±2 at %), while for samples with a greater Rh bulk content, the results of EDS analyses give a systematically smaller Rh content (by up to ca. −18 at % for a sample containing ca. 32% Rh in the bulk, according to EDS data) than that obtained from AES measurements. Since the latter technique is more local and the information depth is much shallower than for both XPS and EDS, the differences in compositions may originate from a real effect of subsurface layer enrichment with Pd.

In the case of Ru alloys, AES could not have been used, due to a strong overlapping of the signals originating from Ru with those from Pd and also from carbon contaminations, which are always detected on solid surfaces [74].

The compositions of Pd-based electrodes were also determined by inductively coupled plasma atomic emission spectroscopy (ICP-AES). For that purpose, after the electrochemical measurements, the samples were dissolved in aqua regia, and the obtained solutions were analyzed. This technique provides data on the total composition of the deposit, averaged over its entire volume, together with the information on the total amounts of each element in a given sample. The fact that atomic emission spectroscopic analysis is completely destructive and can be performed only once for each sample during the research procedure are the main disadvantages of this method. However, its insensitivity to local fluctuations in surface and bulk compositions, together with gaining the whole information on the sample composition, are very favorable characteristics of that analytical technique in the context of the examination of our electrodes.

In Figure 8, alloy compositions determined by EDS and atomic emission spectroscopy techniques are compared. Although the differences in the compositions of the individual samples seem to be scattered within several percent (up to ±8%) and could be ascribed to the experimental errors, one should note that for all Ru-containing alloys studied, there is yet a systematic excess of Ru in most EDX results in relation to atomic emission spectroscopy data. Mean differences between Ru concentrations determined by ICP-AES and EDS for Pd-Ru, Pd-Pt-Ru, and Pd-Rh-Ru systems are between ca. −2 at % and −3.6 at %.

In order to explain the above differences, one should take into account that due to partial overlapping in EDS spectrum, the signals originating from some neighboring elements in the periodic table, the separation of the contributions from those elements is difficult and can lead to a systematic error. A known example concerns Pd-Rh alloys [75,76], where Rh content may be overestimated on the basis of EDS spectra in the case of an insufficient resolution of the detector.

Since Ru characteristic X-ray lines are also placed very close to Pd lines, Ru overstating is expected for EDS analysis of Pd-Ru samples. Therefore, due to partly distorted EDS data, the actual differences between the compositions of Pd-Ru and Pd-Pt-Ru electrodes determined by ICP-AES and EDS may be smaller. By the same reason, the depletion with Rh or Ru within the subsurface layers, mirrored in XPS and AES data, as compared with Rh and Ru contents in the entire bulk, may also be less significant than could be suggested by a simple comparison of XPS, AES, and EDS results.

### 3.3. Electrochemical Characterization

Pd-based deposits were characterized electrochemically under the conditions of a cyclic voltammetric experiment in 0.5 M H_2_SO_4_ solutions at room temperature (298 K). The examples and detailed description of cyclic voltammograms recorded for various Pd-based electrodes were presented earlier and will not be repeated here. We want to focus on the possibility of utilizing the cathodic current signal due to surface oxide reduction for alloy surface composition determination.

**Figure 9:**
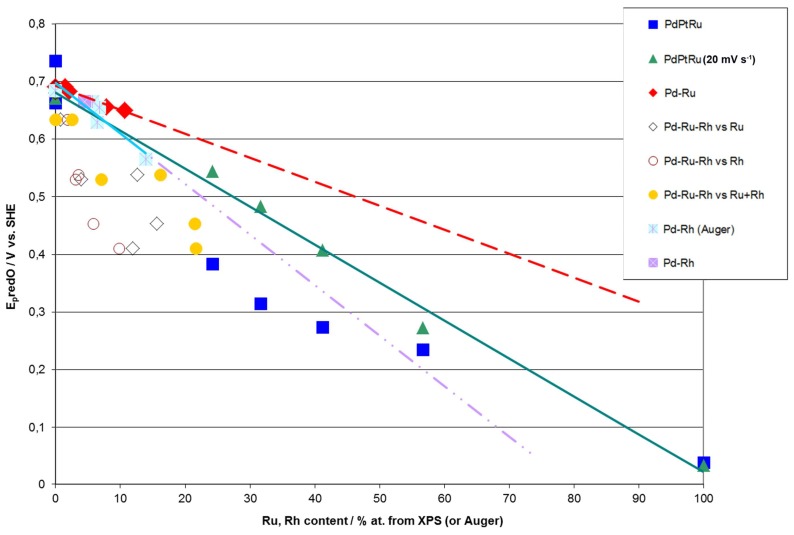
Correlations between the potential of surface oxide reduction peak (E_p_) and metal contents in Pd alloys determined by XPS or AES analyses. Cyclic voltammetry (CV) curves recorded in 0.5 M H_2_SO_4_ solutions at scan rate 100 mV·s^−1^ (and also 20 mV·s^−1^, as indicated for a series for Pd-Pt-Ru electrodes) [58,78].

According to Rand and Woods [77], the potential of surface oxide reduction peak can be recalculated into the surface composition of homogenous binary noble metal alloys (Pt-Rh, Pd-Pt, Pd-Rh and Pd-Au). Due to the fact that the specific electrochemical behavior of a given electrode in surface reactions is determined by its surface properties, we have attempted to verify whether there is a correlation between the potential of the surface oxide reduction peak and the compositions obtained by XPS or AES for Pd-Rh, Pd-Ru, Pd-Pt-Ru, and Pd-Rh-Ru electrodeposits (Figure 9).

Figure 9 demonstrates that for Pd-Rh and Pd-Ru binary systems, good correlations are observed between E_p_ and Rh or Ru contents determined by AES or XPS. However, one should note that although in both cases the variation of E_p_ with metal concentration follows approximately a straight line, the slopes are different than those predicted on the basis of Rand and Woods’ relationship, i.e., these lines extrapolated to 100% of Rh or Ru, do not point at the values of E_p_ measured for those pure metals. This suggests that the surface compositions of Pd-Rh and Pd-Ru electrodes, mirrored in CV profiles, are not identical to the compositions of the subsurface regions of the samples, affecting AES/XPS results.

Although in the case of ternary alloys the potential of surface oxide reduction peak cannot be unequivocally converted into alloy surface composition, it is still possible to use this parameter as a convenient measure of surface concentrations of alloy components or even estimate the surface composition, provided that certain assumptions are made. Figure 9 shows that for Pd-Pt-Ru electrodes, E_p_ tends to decrease with increasing Ru content derived from XPS. Due to the fact that E_p_ for pure Ru is considerably lower than E_p_ for pure Pd and Pt, it is possible to estimate Ru surface content on Pd-Pt-Ru. However, one should bear in mind that within this approximation, the exact position of the surface oxide reduction peak on the potential axis is also affected by the relative proportions between all alloy components, e.g., the same Ru surface concentrations can be accompanied by different Pd and Pt concentrations. Therefore, the values of E_p_ may be different for electrodes containing the same amounts of Ru, and vice versa, the same values of E_p_ may be observed for the electrodes with different Ru contents.

On the other hand, even such a rough approximation fails for the Pd-Rh-Ru system, as E_p_ for Ru and Rh differs by ca. 300 mV and a similar difference occurs between E_p_ for Rh and Pd. The correlation of E_p_ vs. Ru and/or Rh content is now worse than in the case of the systems described above, although in general, E_p_ still tends to decrease with increasing concentration of Ru and Rh, as well as their sum on the electrode surface and in subsurface regions (XPS data).

**Figure 10:**
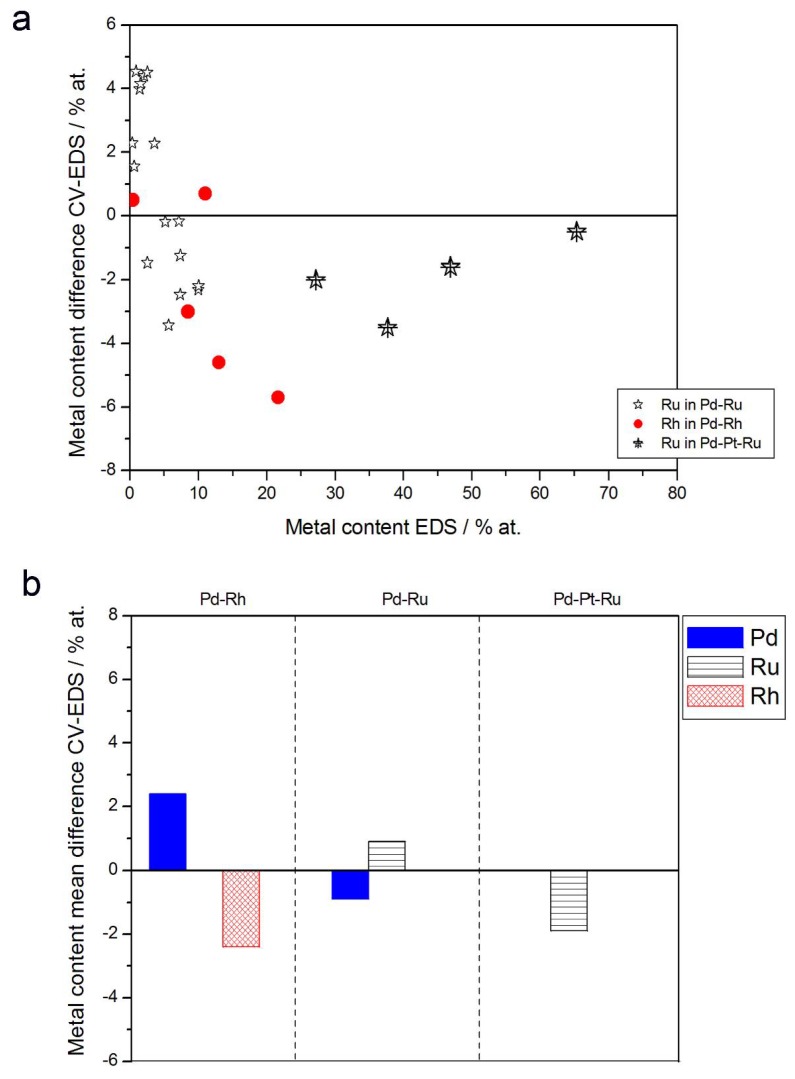
Comparison of the results of quantitative CV and EDS analyses of Pd alloys with Ru, Rh, and/or Pt. (**a**) Difference between metal concentrations determined by CV and EDS versus EDS-derived concentrations for individual samples. (**b**) Mean difference between metal concentrations determined by CV and EDS for each type of alloy.

**Figure 11:**
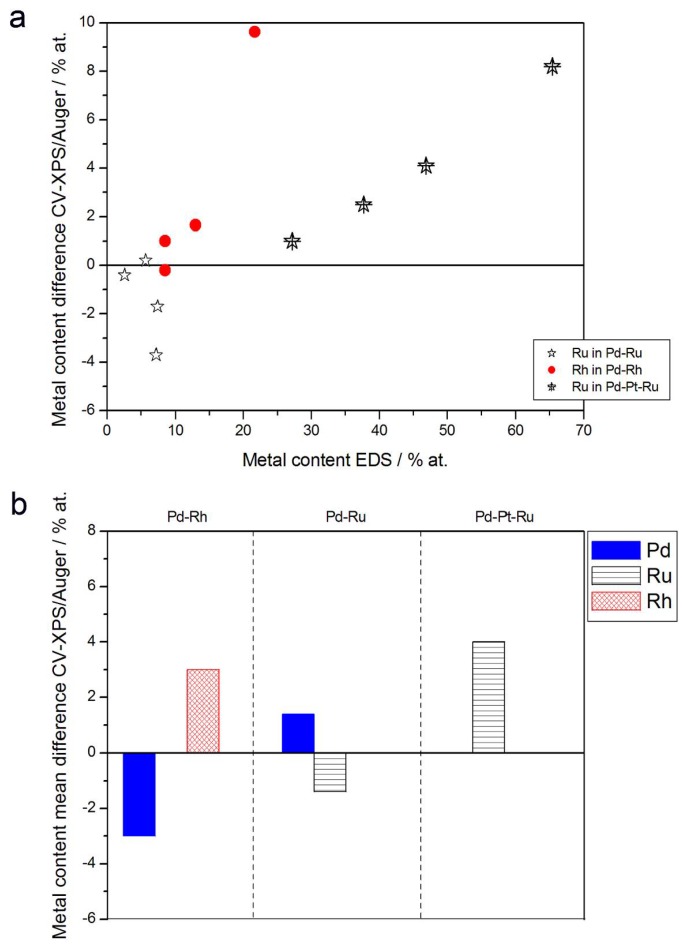
Comparison of the results of quantitative CV and XPS or AES analyses of Pd alloys with Ru, Rh, and/or Pt. (**a**) Difference between metal concentrations determined by CV and XPS/AES versus EDS-derived concentrations for individual samples. (**b**) Mean difference between metal concentrations determined by CV and XPS/AES for each type of alloy.

Figure 10 and Figure 11 show a comparison of alloy compositions determined by CV and EDS or XPS/AES. The following observations can be made:

(1) Rh content determined by EDS is usually higher than those derived from XPS or AES measurements, and at the same time Rh content originating from AES data is higher than that from XPS. For Pd-Rh samples in the composition range examined in this study, Rh concentration determined by CV is similar to or lower than that obtained by EDS (differences up to −5.7 at % for the individual samples, and ca. −2.4 at % on average). This is in line with our earlier data on the electrochemistry of the Pd-Rh system [79,80]. However, according to the results of the experiments performed with Pd-Rh electrodes in a much wider composition range, for Pd-Rh electrodeposits containing more than ca. 20% Rh in the bulk, the surface becomes more and more enriched with Rh [79,80];
(2) For individual Pd-Ru samples, the differences in Ru contents determined by CV and EDS vary within the range ±4 at %, but the mean differences do not exceed +1 at %;
(3) For Pd-Pt-Ru electrodes, the analysis of CV curves gives a systematically smaller Ru content with respect to EDS data (to −3.5 at % for the individual samples and ca. −2 at % on average);
(4) The differences in Ru or Rh contents determined by CV and XPS/AES are opposite to CV vs. EDS-derived values. In particular, for Pd-Pt-Ru alloys with increasing bulk Ru content (>25%), more Ru (by up to +8 at % individually and ca. +4 at % on average) is detected by CV than by XPS, while for the same samples, CV data are closer to EDS data. A similar situation occurs for CV vs. AES data for Pd-rich Pd-Rh alloys, while for Pd-rich Pd-Ru alloys CV analysis gives a smaller Ru content (to −4 at % individually and ca. −1.5 at % on average) than that based on XPS analysis.

It should be noted that the Pd-Rh system is particularly convenient for studying the relations between alloy compositions on different orders of layer thickness, because it is possible to apply the CV, AES, and XPS techniques to determine surface and subsurface metal contents in Pd-Rh electrodes. For other Pd-based systems studied here, there is always a lack of data for at least one method, due to the limitations described above.

### 3.4. Comparison of Data Obtained by Various Analytical Techniques

**Figure 12:**
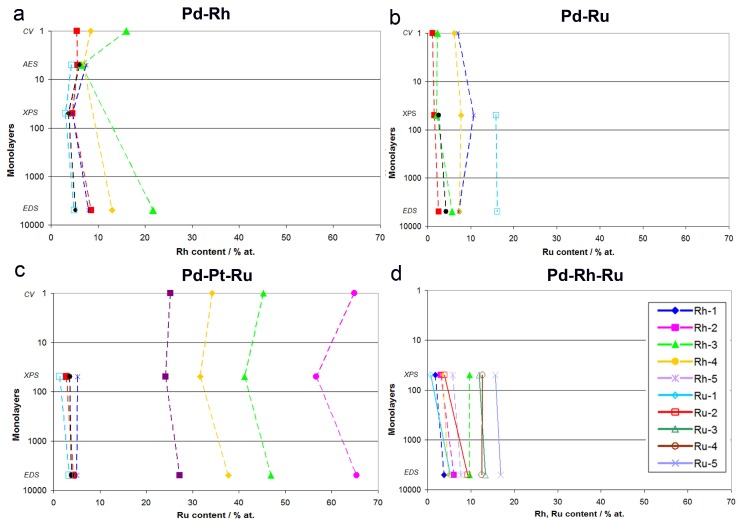
Comparison of alloy compositions obtained by various analytical techniques in relation to the information depth characteristic of each technique: (**a**) Pd-Rh, (**b**) Pd-Ru, (**c**) Pd-Pt-Ru, (**d**) Pd-Rh-Ru.

Figure 12 shows a summary of the results of CV, AES, XPS, and EDS analyses of Pd-Rh, Pd-Ru, Pd-Rh-Ru, and Pd-Pt-Ru alloys. For each system, the compositions obtained by a particular technique are related to the maximum information depth characteristic of that technique, expressed as a number of monolayers for fcc crystal structure (assuming that one unit cell corresponds to two atomic layers). A given analytical method provides information on the sample composition averaged over all layers above its maximum information depth, being insensitive to the composition of the layers lying underneath. Namely, CV probes the most outer atomic layer, AES analysis reaches down to ca. 5 monolayers (1 nm), XPS to ca. 50 monolayers (10 nm), and EDS to ca. 5000 monolayers (1 μm). Thus, AES analytic signal contains information provided by CV, and additionally that originating from ca. 4 lower atomic layers, while in XPS data, both CV and AES results are included, however, together with the extra information on the composition of tens of deeper atomic layers not probed by CV/AES. EDS collects data from the largest portion of the sample, and its signal is dominated by the bulk of the material analyzed, although it still contains information from the surface and subsurface layers.

Summarizing the results presented in Figure 12 and in earlier paragraphs, one should note the aforementioned various information depths offered by various techniques, together with their various lateral and volumetric resolutions. When comparing CV and AES data for Pd-Rh alloys, the observed differences may be ascribed to the fact that the course of a CV curve mirrors the electrochemical processes of surface oxide reduction involving the first monolayer, or even a submonolayer of the sample, while the AES signal originates from ca. 5 external atomic layers. Therefore, the information contained in the AES signal is affected not only by the composition of the upmost monolayer, but also by the composition of ca. 4 underlying layers, which may contain different amounts of the metals. Their contribution may dominate over the contribution from the very first layer, resulting in a discrepancy between CV and AES data. On the other hand, one should also take into account the variations in peak potential within the range of the order of 10 mV, often occurring in CV experiments between consecutive cycles, due to changes in surface state caused by the electrodissolution of metals [77]. This potential shift corresponds to the uncertainty in alloy surface composition within the range of ca. 4 at %, which is comparable with the difference between CV and XPS or AES data observed for Pd-rich (>85% Pd in the bulk) Pd-Rh samples studied.

The relation of CV vs. XPS results is supposed to be affected much more by the average composition of the subsurface layers than the relation of CV vs. AES data. The highest differences between both sets of data are observed in the case of Pd-Pt-Ru electrodes containing more than 65% Ru in the bulk, however, these deviations do not individually exceed +8.5 at %, while for Pd-rich (>90% in the bulk) Pd-Ru alloys, they are less significant than −4 at %.

On the other hand, the differences between CV and EDS results can be treated as a true relation between surface and bulk compositions of the deposits. For Pd-Ru and Pd-Rh samples rich in Pd (more than ca. 80% in the bulk), Ru and Rh surface contents generally do not deviate markedly from their bulk contents. In that context, we have to explain that in our earlier report [78] we estimated the surface compositions of Pd-Ru electrodeposits on the basis of CV data, using the dependence between the potential of surface oxide reduction peak and Ru surface content calibrated on XPS results. However, we are aware of the limitations of such an approximation, due to the fact that the information depth in the XPS method is greater than that corresponding to the most external surface layer, and therefore the composition of some underneath layers also affects the XPS signal. Unfortunately, a reliable quantitative AES, certainly desired here, was not possible to perform for the Pd-Ru system.

An interesting picture emerges from a comparison of XPS and EDS data, as it concerns the relation of subsurface vs. bulk compositions of the alloys studied. A careful inspection of Figure 12 reveals that there are some distinct differences between both series of results, even greater than those between CV and XPS/AES or EDS data. In particular, the highest discrepancies between XPS and EDS data are observed for ternary systems, where the concentrations of one or two components (i.e., Pd in Pd-Rh-Ru alloys, Pt and Ru in Pd-Pt-Ru alloys) determined by XPS deviate markedly from those determined by EDS. This observation suggests that the subsurface regions of these alloys possess some individual properties regarding the elements distribution in comparison with the interior of the sample, as well as with the most external layers lying above.

One should note that, taking into account the information depth characteristic of XPS, i.e., ca. 10 nm, and the overall thickness of the electrodeposits, i.e., ca. 0.5–1.0 μm, this subsurface region corresponds to ca. 1–2% of the entire sample thickness. Thus, this region cannot be treated as a separate alloy phase, and its presence does not contradict the conclusion that the fresh electrodeposits obtained from chloride baths are usually highly homogeneous in the bulk. In that context, it is noteworthy to mention that phase diagrams of Pd alloys with Rh or Ru are characterized by the presence of a miscibility gap at intermediate concentrations of the metals. Therefore, our samples not subjected to any form of pretreatment are probably metastable at room temperature, and the conditions of thermodynamic equilibrium required for phase separation are not fulfilled. The conclusion on the homogeneity of Pd-rich electrodeposits was confirmed in many experiments concerning hydrogen electrosorption in those materials [76,78,79]. However, it may be curious to mention here that the thickness of the subsurface region corresponding to XPS information depth coincides with the electrode thickness, where subsurface hydrogen is postulated to exist during the hydrogen absorption/desorption processes [81,82].

## 4. Conclusions

Pd-rich binary and ternary codeposits with Pt, Rh, and Ru, obtained by potentiostatic deposition or by chemical co-precipitation of metals from chloride solutions, form face-centered cubic (fcc) substitutional solid solutions, with lattice parameters smaller than that of pure Pd (contracted alloys). Pd-rich alloys are highly homogeneous within the entire bulk and on the surface. The samples rich in Ru and/or Rh may segregate into separated phases, including those of a lattice symmetry different than fcc. 

Electrodeposition enabled us to prepare Pd-Rh, Pd-Ru, Pd-Pt-Ru, and Pd-Rh-Ru alloys in a wide range of compositions, like a series of Pd-Rh (>45% Pd in the bulk) and Pd-Ru (>90% Pd in the bulk) alloys investigated in the context of hydrogen electrosorption or electrocatalysis. Before our work, the Pd-Rh-Ru system has not been obtained in the form of electrodes, nor studied electrochemically. Some other methods described in the literature, such as volume melting, powder sintering, or chemical reduction, often do not allow one to obtain homogeneous samples, especially for Ru- or Rh alloys.

In general, Pd-rich alloys are characterized by very similar bulk and surface compositions, while for alloys rich in Ru and/or Rh in the bulk, a surface excess of these elements is observed. On the other hand, usually less Ru and/or Rh was detected by XPS than by CV, AES, or EDS techniques. Taking into account that CV merely provides information from the surface, AES examines a greater thickness (ca. 1 nm), XPS probes deeper (ca. 10 nm), and EDS in fact provides information on a much larger depth (0.5–1 μm), our Pd electrodeposits exhibited the greatest differences in the composition of subsurface layers with respect to both the mere surface and the bulk.

The comparison between the properties of Pd-Pt-Ru electrodeposits on Au and Pd-Pt-Ru/VulcanXC72 chemical deposits shows that, despite marked differences in surface morphology of both kinds of samples, their crystallographic structures are relatively similar, but not identical. For the electrodeposited alloys, a higher Ru bulk content was accompanied by a smaller crystallite size, and by a higher inhomogeneity between the core and the surface of the crystallites, while the crystallites of the Pd-Pt-Ru/VulcanXC72 chemical co-precipitates were ca. twice greater in size, but more homogeneous, even for the alloys with a high Ru bulk content.

The results obtained by the spectroscopic and electrochemical techniques are mutually consistent, and all methods of solid sample characterization are often complementary. According to a general rule of modern material science and chemical analysis, the most complete attainable characterization of physical and chemical properties of a solid requires the utilization of several microscopic, spectroscopic, and structural techniques probing different volumes, depths, and area sizes of the sample, with different lateral resolutions and different possibilities of local or global analysis.
